# Dissecting the current caesarean section rate in Shanghai, China

**DOI:** 10.1038/s41598-019-38606-7

**Published:** 2019-02-14

**Authors:** Yanhong Ming, Meng Li, Fei Dai, Rong Huang, Jinwen Zhang, Lin Zhang, Ming Qin, Liping Zhu, Hongping Yu, Jun Zhang

**Affiliations:** 1 Department of Obstetrics and Gynecology, Xiangya Hospital, Central South University, Changsha, Hunan 410008 China; 2 School of Public Health, Guilin Medical University, Guilin, Guangxi 541004 China; 3 Ministry of Education-Shanghai Key Laboratory of Children’s Environmental Health, Xinhua Hospital, Shanghai Jiao Tong University School of Medicine, Shanghai, 200092 China; 4 School of Public Health, Shanghai Jiao-Tong University School of Medicine, Shanghai, 200025 China; 5 Xinhua Hospital, Shanghai Jiao Tong University School of Medicine, 1665 Kongjiang Road, Shanghai, 200092 China; 6 Shanghai Maternal and Child Health Center, Shanghai, 200062 China; 7 Affiliate Cancer Hospital of Guangxi Medical University, Nanning, Guangxi 530021 China; 8 Department of Epidemiology, School of Public Health, Guangxi Medical University, Nanning, Guangxi 530021 China

## Abstract

The high caesarean section (CS) rate has been of great public concern around the world. Yet, large-scale studies of dissecting such a high CS rate are few in the Chinese population. We carried out a cross-sectional survey randomly selecting 10,855 births from 20 hospitals in Shanghai from January to June, 2016. Labor and delivery information was extracted from medical records. The Robson classification system for CS was used to classify all women into ten groups. The overall CS rate was 41.5%. Prelabor CS in nulliparous, term singleton vertex women was the predominant contributor (37.4%) to the total CS and accounted for the second highest proportion of total births (15.5%) in all hospital types. The vast majority of women with a previous CS had a repeat CS (96.6%). CS rate was still high in Shanghai. Nulliparous women in low risk and having CS before labour, often without any medical indication, was a major contributor to the high CS rate.

## Introduction

The cesarean section (CS) rate has been rising worldwide over the last two to three decades^1^. A recent large scale study showed that the overall CS rate in China increased from 28.8% in 2008 to 34.9% in 2014^2^. However, the CS rates in urban settings of China are much higher than the national average and have changed in a different pattern. For example, in Shanghai, the CS rate reached its peak at 60.9% in 2008 and declined to 50.8% in 2014^2^. With the adoption of two-child family policy in China, how the CS rate may change remains unclear. On one hand, nulliparous women may be more inclined than before to have a vaginal birth with consideration of having a second child later. On the other hand, the very high CS rate in earlier years had resulted in a high proportion of multiparous women with a scarred uterus. Repeat CS may even increase the overall CS rate in the near future. However, large obstetric databases with reliable and sufficient details are still rarely available in most parts of China to understand the variation in CS rate among hospitals and the causes for a very high overall CS rate.

The Robson Ten-Group Classification System (RTGCS) offers a useful tool to dissect the overall CS rate and facilitate the understanding of the components. It classifies all deliveries into one of ten groups based on five basic parameters^3^: obstetric history, onset of labor, fetal lie, number of fetuses, and gestational age. The RTGCS is a simple and reliable delivery classification system that has gained wide acceptance by the international obstetric and midwifery community^4^. It also enables comparisons between different districts and institutions. In this study, we used RTGCS to classify pregnant women into subgroups and compared the CS rate by different hospital types and subgroups. Due to the important role of birth weight in mode of delivery^5^, we also analyzed the mode of delivery according to birth-weight category by RTGCS. Findings of this study may help develop strategies to reduce CS rate in China.

## Materials and Methods

### Study design and study population

**Figure 1:**
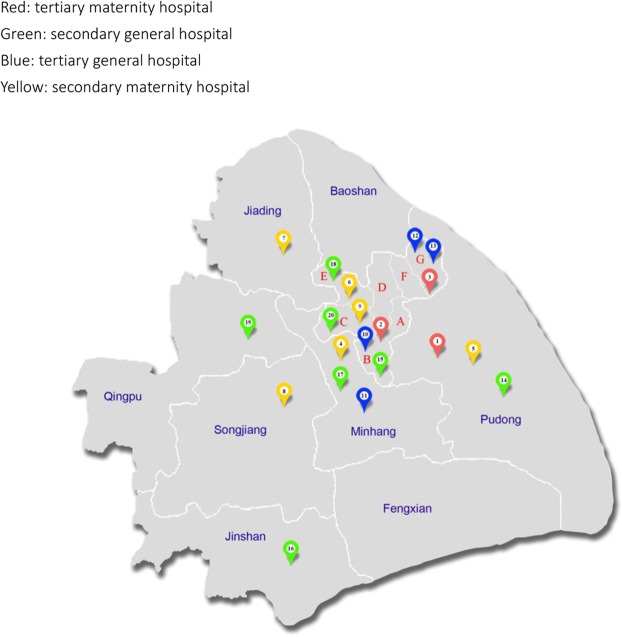
Distribution of the 20 hospitals in Shanghai. *Red: tertiary maternity hospitals; Green: secondary general hospitals; Blue: tertiary general hospitals; Yellow: secondary maternity hospitals.

There are 79 hospitals that provide delivery services in Shanghai right now. We selected top 20 hospitals based on the annual delivery volume, including 3 tertiary maternity hospitals, 6 secondary maternity hospitals, 4 tertiary general hospitals and 7 secondary general hospitals across Shanghai in 2016 (Fig. 1). Primary care hospitals were not included in the present study, as they usually do not provide obstetric services. These hospitals delivered approximately half of all births in Shanghai. A total of 62,653 births were delivered in these hospitals from January 1 2016 to June 30 2016. Since the number of annual deliveries in each hospital varied greatly, from around 1,000 to nearly 20,000 births a year, to ensure the precision of the CS rate estimates in each hospital, in hospitals that had an annual delivery volume below 10,000 births, 20% of the total births were randomly selected while in hospitals with more than 10,000 births a year, 10% of the total births were randomly selected. In each hospital, a list of all eligible deliveries within the study period was generated from the hospital information system. A predetermined percentage of records were randomly selected by using computer-generated random digits. Either electronic or paper medical records were retrieved and relevant information was extracted. A total of 10,855 deliveries were selected. These deliveries represented the total births during that period. A weight using an inverse probability weighting method was assigned to each woman.

Trained medical students carried out the data abstraction. Detailed information on maternal demographic characteristics, prenatal history, labor and delivery, and neonatal conditions was recorded. To make our findings comparable to other studies, we restricted the analysis to births more than 24 gestational weeks or birth weight more than 500 g, including all live births, stillbirth and fetal deaths and second trimester abortion. 48 women with missing medical records, 41 women with second trimester abortion, 14 women without the mode of delivery and 1 woman without neonatal birth weight were excluded from analysis. In total, 10,751 women were included.

### Analysis and statistical methods

Women were categorized into 10 groups according to the RTGCS. For each group, the CS rate (the number of CS divided by total number of deliveries), the percent of total births (total deliveries in each group divided by total deliveries), and the proportion of total CS (CS in each group divided by total number of CS) were calculated. The database was built in EPI data 3.0, and all analyses were conducted using SPSS statistics software version 22.0 (IBM, Armonk, NY, USA). This study is a descriptive analysis in a large sample, thus neither other statistical testing nor confidence intervals were provided excepting Mantel-Haenszel Chi-Square test for analyzing mode of delivery according to birth-weight category by Robson Classification System. This study was approved by the Ethics Committees of Xinhua Hospital and all participating hospitals. No individual consent was required for chart abstraction of deidentified information. All research was performed in accordance with relevant guidelines or regulations.

### Ethics approval

This study has been approved by the Ethics committee of Xinhua Hospital Affiliated to Shanghai Jiao Tong University School of Medicine, Shanghai, China. (protocol no. XHEC-C-2016-095).

## Results

Among the 20 hospitals, 11 were located in the city proper (Fig. 1). 12 hospitals delivered fewer than 5,000 births; 4 delivered between 5,000 and 10,000 births; and 4 had more than 10,000 deliveries in 2016. Table 1 presents the basic characteristics of the subjects by hospital type. Nearly 80% of women were 25 to 35 years old and 54.2% had medical insurance. Seven in ten pregnant women had a college or higher degree, and office worker (60.2%) was the most common occupation, followed by unemployed (25.8%) and researcher/teacher/doctor (10%). Nearly sixty-five percent of pregnant women were nulliparous; 13.9% were with previous CS; 95.6% had cephalic presentation, and 1.7% were multiple gestations. The average prepregnancy body mass index (BMI) of the women was 21.6 ± 3.3 kg/m^2^, with underweight, normal weight, overweight and obese women accounting for 15%, 51.4%, 16.9%, and 16.8%, respectively (based on WHO 2000 Asian BMI cut points)^6^. Pre-pregnancy hypertension, diabetes, heart disease and kidney disease were found in 0.6%, 0.2%, 1.0% and 0.2% of women, respectively.

**Table 1:** The basic characteristics of women by hospital type in Shanghai.

Characteristics	Tertiary maternity hospitals (n = 2013)	Secondary maternity hospitals (n = 3487)	Tertiary general hospitals (n = 1741)	Secondary general hospital (n = 3510)	Total (n = 10751)
Number of hospitals per type	3	6	4	7	20
Age of delivery (year, mean ± SD)	30.9 ± 3.8	29.5 ± 4.0	29.7 ± 4.5	28.4 ± 4.7	29.4 ± 4.3
Age group (%)					
<20	0.1	0.3	1.2	1.6	0.8
20-	2.4	8.5	9.1	17. 7	10.4
25-	38.5	45.5	43.4	45.3	43.8
30-	41.9	34.2	32.3	24.6	32.2
35-	17.1	11.4	14.1	10.7	12.7
Missing	0	0.1	0	0.1	0.1
Education (%)					
Less than high school	3.6	11.6	3.9	34.9	14.5
High school	7.0	18.3	7.5	24.6	14.6
College or University	72.6	65.6	76.5	38.8	61.7
Postgraduate	16.9	4.5	12.2	1.7	9.1
Occupation					
Physical worker	0.1	0.6	10.7	8.1	4.0
Officer worker	76.3	65.1	60.1	40.4	60.2
Researcher, teacher, doctor, *et al*.	8.4	9.1	18.0	8.2	10.0
Unemployed (including students)	15.2	25.2	11.1	43.3	25.8
Medical insurance (%)	64.5	64.1	58.9	36.3	54.2
Nulliparity	78.2	66.9	67.2	53.2	64.6
Previous C-section	9.8	12.4	13.2	18.1	13.9
Multiple pregnancy	3.6	1.1	2.1	1.0	1.7
Presentation					
Cephalic	95.1	95.4	95.4	96.1	95.6
Breech	4.8	4.3	4.2	3.7	4.2
Other/transverse lie	0.2	0.3	0.4	0.2	0.2
*BMI (kg/m^2^, mean ± SD)	21.1 ± 3.1	21.4 ± 3.2	21.7 ± 3.3	22.1 ± 3.4	21.6 ± 3.3
Underweight (<18.5 kg/m^2^, %)	17.8	16.4	14.4	12.3	15.0
Normal weight (18.5–22.9 kg/m^2^, %)	55.6	53.3	50.8	47.0	51.4
Overweight (23–24.9 kg/m^2^, %)	14.9	15.6	17.2	19.2	16.9
Obese (0 ≥ 25 kg/m^2^, %)	11.8	14.7	17.6	21.6	16.8
Disease history (yes%)					
Hypertension (%)	1.4	0.1	0.8	0.4	0.6
Diabetes (%)	0.3	0.1	0.3	0.1	0.2
Heart disease (%)	2.7	0.6	1.0	0.5	1.0
Kidney disease (%)	0.2	0.2	0.7	0.1	0.2

^*^Source: weight status was defined according to WHO Asian BMI cut points^5^.

Table 2 shows the indications of CS in different hospital types in Shanghai. The top six indications were repeat CS, fetal distress, patient request, non-cephalic fetal presentation, suspected macrosomia, and cephalopelvic disproportion, accounting for 76% of all CS. The rest of indications and unknown indications accounted for 23.7% and 0.3%, respectively (Detailed list of indications are listed in Appendix). Repeat CS was the first CS indication in all types of hospitals, accounting for 20.4%, 32.0%, 26.2% and 34.9% of the total CS, respectively. The order and contribution of other indications varied among the hospital types. For example, patient request was the number two reason in the secondary maternity hospital, accounting for 13.9% of the total CS while fetal distress was the number two reason all other types of hospitals, accounting for 18.1%, 16.1%, and 21.0% of the total CS, respectively.

**Table 2:** Indications of caesarean section in different hospital types in Shanghai.

Indications of CS	Total				Tertiary maternity Hospital			Secondary maternity Hospital			Tertiary general Hospital			Secondary general Hospital		
	N	Absolute CS rate	Proporti-on of total CS	Order	Absolute CS rate	Proportion of total CS	Order	Absolute CS rate	Proportion of total CS	Order	Absolute CS rate	Proportion of total CS	Order	Absolute CS rate	Proportion of total CS	Order
1. Repeat CS	7764	12.4	29.8	1	8.3	20.4	1	12.9	32.0	1	12.6	26.2	1	15.3	34.9	1
2. Fetal distress	4354	7.0	16.7	2	7.3	18.1	2	4.6	11.5	3	7.8	16.1	2	9.2	21.0	2
3. Patient request	2770	4.4	10.7	3	3.9	9.6	4	5.6	13.9	2	5.2	10.8	3	3.6	8.2	4
4. Non-cephalic fetal presentation	2476	4.0	9.5	4	4.4	11.0	3	4.4	11.0	4	3.8	7.8	4	3.7	8.4	3
5. Suspected macrosomia	1405	2.2	5.4	5	2.4	6.0	5	2.4	5.9	5	2.6	5.3	5	2.1	4.7	6
6. Cephalopelvic disproportion	1008	1.6	3.9	6	0.2	0.5	16	1.8	4.4	6	1.1	2.3	9	2.7	6.1	5

Table 3 shows the CS rate and proportion in subgroups by RTGCS. Nulliparous, term singleton vertex women in spontaneous labour (NS group) accounted for the highest proportion of total births (30.7%), followed by nulliparous, term singleton vertex women with CS before labour (NC group) (15.5%). Term, singleton, vertex women with previous CS (PC group) accounted for 11.1% of total birth. The majority of pregnant women (96.6%) with a previous CS (PC group) had repeat CS. Nulliparous, term singleton vertex women in spontaneous labor (NS groups) had a relatively lower total CS rate (6.9%) than in induced labour (NI groups) (19%). Nulliparous, term singleton vertex women with CS before labour (NC group) was the predominant contributor to the total CS (37.4%), followed by term singleton vertex women with previous CS (PC group, 25.9%).

**Table 3:** The cesarean section rate and proportion in subgroup by Robson Ten-Group Classification System.

Robson Classification	Characteristics	Percent of total births (%)	CS rate (%)	Proportion of total caesareans (%)
NS (1)	Nulliparous, singleton, cephalic, ≥37 weeks’ gestation, in spontaneous labour	30.7	6.9	5.1
NI (2a)	Nulliparous, singleton, cephalic, ≥37 weeks’ gestation, induced labour	13.7	19.0	6.3
NC (2b)	Nulliparous, singleton, cephalic, ≥37 weeks’ gestation, caesarean section before labour	15.5	100.0	37.4
MS (3)	Multiparous (excluding previous caesarean section), singleton, cephalic, ≥37 weeks’ gestation, in spontaneous labour	12.4	2.2	0.7
MI (4a)	Multiparous without a previous uterine scar, with singleton, cephalic pregnancy, ≥37 weeks’ gestation, induced labour	2.6	4.3	0.3
MC (4b)	Multiparous without a previous uterine scar, with singleton, cephalic pregnancy, ≥37 weeks’ gestation, caesarean section before labour	1.4	100.0	3.3
PC (5)	Previous caesarean section, singleton, cephalic, ≥37 weeks’ gestation	11.1	96.6	25.9
BR (6 + 7 + 9)	All nulliparous with a single breech, All multiparous with a single breech (including previous caesarean section), and All women with a single pregnancy in transverse or oblique lie (including those with previous caesarean section)	4.5	94.1	10.1
TW (8)	All multiparous (including previous caesarean section),	2.2	94.1	5.0
PT (10)	All singleton, cephalic, <37 weeks’ gestation pregnancies (including previous caesarean section)	5.3	44.1	5.6
UK (99)	Unknown	0.5	21.6	0.3
	Total	100.0	41.5	100.0

Table 4 further shows the CS rate and proportion in different hospital types by RTGCS. The tertiary maternity hospital had the lowest CS rate in nulliparous, term singleton vertex women in spontaneous labor (NS groups) (3.13%) and in induced labor (NI group) (13.6%), but accounting for the highest contributor to the total CS in nulliparous, term singleton vertex women with CS before labour (NC group) (44.6%). Otherwise, CS rate in term singleton vertex women with a previous CS (PC group) was also the lowest in the tertiary maternity hospital than other types of hospitals (94.21%).

**Table 4:** The cesarean section rate and proportion in different hospital type by Robson Ten-Group Classification System.

Robson Classification	Characteristics	Hospital type	Percent of total births (%)	CS rate (%)	Proportion of total caesareans (%)
NS (1)	Nulliparous, singleton, cephalic, ≥37 weeks’ gestation, in spontaneous labour				
		Tertiary maternity Hospital	32.86	3.13	2.54
		Secondary maternity Hospital	31.25	8.17	6.37
		Tertiary general Hospital	29.9	10.97	6.83
		Secondary general Hospital	23.5	13.37	7.15
NI (2a)	Nulliparous, singleton, cephalic, ≥37 weeks’ gestation, induced labour				
		Tertiary maternity Hospital	16.07	13.6	5.4
		Secondary maternity Hospital	13.35	21.51	7.16
		Tertiary general Hospital	10.05	33.07	6.92
		Secondary general Hospital	10.95	22.98	5.73
NC (2b)	Nulliparous, singleton, cephalic, ≥37 weeks’ gestation, caesarean section before labour				
		Tertiary maternity Hospital	18.05	100	44.6
		Secondary maternity Hospital	13.85	100	34.55
		Tertiary general Hospital	17.09	100	35.59
		Secondary general Hospital	12.07	100	27.47
MS (3)	Multiparous (excluding previous caesarean section), singleton, cephalic, ≥37 weeks’ gestation, in spontaneous labour				
		Tertiary maternity Hospital	7.87	1.53	0.3
		Secondary maternity Hospital	14.83	2.37	0.88
		Tertiary general Hospital	12.06	3.78	0.95
		Secondary general Hospital	18.37	1.93	0.81
MI (4a)	Multiparous without a previous uterine scar, with singleton, cephalic pregnancy, ≥37 weeks’ gestation, induced labour				
		Tertiary maternity Hospital	2.07	0	0
		Secondary maternity Hospital	2.91	3.68	0.27
		Tertiary general Hospital	1.7	6.75	0.24
		Secondary general Hospital	4.11	11.23	1.05
MC (4b)	Multiparous without a previous uterine scar, with singleton, cephalic pregnancy, ≥37 weeks’ gestation, caesarean section before labour				
		Tertiary maternity Hospital	0.58	100	1.43
		Secondary maternity Hospital	1.64	100	4.1
		Tertiary general Hospital	2.19	100	4.57
		Secondary general Hospital	2.34	100	5.33
PC (5)	Previous caesarean section, singleton, cephalic, ≥37 weeks’ gestation				
		Tertiary maternity Hospital	8.51	94.21	19.82
		Secondary maternity Hospital	11.75	98.68	28.93
		Tertiary general Hospital	11.18	97.96	22.82
		Secondary general Hospital	16.76	94.68	36.13
BR (6 + 7 + 9)	All nulliparous with a single breech, All multiparous with a single breech (including previous caesarean section), and All women with a single pregnancy in transverse or oblique lie (including those with previous caesarean section)				
		Tertiary maternity Hospital	4.27	97.93	10.34
		Secondary maternity Hospital	4.53	98	11.08
		Tertiary general Hospital	5.02	87.33	9.13
		Secondary general Hospital	4.48	76.29	7.79
TW (8)	All multiparous (including previous caesarean section)				
		Tertiary maternity Hospital	3.73	95.45	8.79
		Secondary maternity Hospital	1.16	93.8	2.7
		Tertiary general Hospital	2.13	83.1	3.68
		Secondary general Hospital	1.08	97.52	2.4
PT (10)	All singleton, cephalic, <37 weeks’ gestation pregnancies (including previous caesarean section)				
		Tertiary maternity Hospital	5.51	49.01	6.68
		Secondary maternity Hospital	4.42	35.07	3.87
		Tertiary general Hospital	7.34	52.42	8.02
		Secondary general Hospital	5.81	44.22	5.85
UK (99)	Unknown				
		Tertiary maternity Hospital	0.48	8.63	0.1
		Secondary maternity Hospital	0.3	14.2	0.11
		Tertiary general Hospital	1.34	44.97	1.25
		Secondary general Hospital	0.51	24.3	0.28

Table 5 shows the mode of delivery according to birth-weight category by RTGCS. The CS rate increased with increasing birth-weight above 2500 g for nulliparous/multiparous, term singleton vertex women in spontaneous labor (NS and MS groups, P < 0.01), and pregnant women with a previous CS (PC group). The CS rate was only 36.7% in neonatal birth-weight of 3000–3499 g, compared with 61.9% in neonatal birth-weight above 4000 g. For each birth-weight category, the CS rate was higher in nulliparous/ multiparous, term singleton vertex women in induced labour (NI/MI group) than in spontaneous labor (NS/MS group).

**Table 5:** Cesarean section rate by birth-weight category in Robson Ten-Group Classification System.

Robson Classification	Characteristics	CS number and CS rate by birth-weight category					
		<2500 g	2500–2999 g	3000–3499 g	3500–3999 g	≥4000 g	P*
		N (%)	N (%)	N (%)	N (%)	N (%)	
NS (1)	Nulliparous, singleton, cephalic, ≥37 weeks’ gestation, in spontaneous labour	5 (4.76)	134 (4.53)	769 (7.80)	641 (12.39)	144 (21.69)	<0.0001
NI (2a)	Nulliparous, singleton, cephalic, ≥37 weeks’ gestation, induced labour	17 (36.96)	151 (14.98)	629 (16.76)	728 (27.28)	227 (44.25)	<0.0001
NC (2b)	Nulliparous, singleton, cephalic, ≥37 weeks’ gestation, caesarean section before labour	134 (100)	985 (100)	3468 (100)	2996 (100)	1377 (100)	N/A^a^
MS (3)	Multiparous (excluding previous caesarean section), singleton, cephalic, ≥37 weeks’ gestation, in spontaneous labour	0 (0)	17 (1.81)	81 (1.96)	69 (2.19)	28 (4.04)	<0.01
MI (4a)	Multiparous without a previous uterine scar, with singleton, cephalic pregnancy, ≥37 weeks’ gestation, induced labour	0 (0)	5 (3.42)	52 (7.08)	46 (6.32)	17 (7.66)	N/A^a^
MC (4b)	Multiparous without a previous uterine scar, with singleton, cephalic pregnancy, ≥37 weeks’ gestation, caesarean section before labour	12 (100)	140 (100)	332 (100)	385 (100)	146 (100)	N/A^a^
PC (5)	Previous caesarean section, singleton, cephalic, ≥37 weeks’ gestation	41 (100)	758 (94.87)	3223 (95.67)	2640 (95.76)	698 (96.68)	0.30
BR (6 + 7 + 9)	All nulliparous with a single breech, All multiparous with a single breech (including previous caesarean section), and All women with a single pregnancy in transverse or oblique lie (including those with previous caesarean section)	186 (93.94)	589 (100)	1096 (99.46)	559 (98.94)	134 (100)	<0.0001
TW (8)	All multiparous (including previous caesarean section),	464 (90.8)	424 (98.6)	69 (85.19)	6 (50)	6 (100)	<0.0001
PT (10)	All singleton, cephalic, <37 weeks’ gestation pregnancies (including previous caesarean section)	528 (41.67)	484 (42.31)	414 (51.36)	116 (58.59)	12 (66.67)	<0.0001
UK (99)	Unknown	5 (29.41)	34 (36.56)	51 (40.16)	34 (30.91)	17 (41.46)	0.55
TOTAL		1392 (58.41)	3721 (40.31)	10184 (36.68)	8220 (43.86)	2806 (61.86)	<0.0001

^*^Mantel-Haenszel Chi-Square test.

^a^Row or column sum zero. No statistics computed for this sub-table.

## Discussion

Our study found that CS rate in Shanghai, China, was still very high, at 41.5%. Nulliparous, term singleton vertex women with CS before labour (NC group) constituted 15.5% of total births and was the largest contributor to the total CS in all hospital types (37.4% overall). The majority of women with previous CS had a repeat CS (96.6%). Nulliparous, term singleton vertex women in induced labour (NI groups) had a relatively higher total CS rate than in spontaneous labor (NS groups) (19% vs 6.9%). The tertiary maternity hospital had the lowest CS rate in nulliparous, term singleton vertex women in spontaneous labor (NS groups) (3.13%) and in induced labor (NI group). The top six indications for CS were repeat CS, fetal distress, patient request, non-cephalic fetal presentation, suspected macrosomia and cephalopelvic disproportion. In nulliparous and multiparous, term singleton vertex women in spontaneous labor (NS and MS groups), and pregnant women with a previous CS (PC group), the CS rate increased consistently with increasing neonatal birth weight above 2500 g.

Nulliparous, term singleton vertex women with CS before labour (NC group) contributed to the most CS deliveries (37.4%), ranging from 27.5% in secondary general hospitals to 44.6% in tertiary maternity hospitals. This proportion is 10 times higher than 3.5% in Netherlands^7^. There are two possible explanations. First, the high proportion of CS in NC group (44.6%) among total CS in tertiary maternity hospitals could be explained by the low CS rate in NS and NI group and consequent low proportion of total CS that they represent. Second, the Chinese government had abolished the “One-Child Family” restriction and permitted the “Two-Child Family” policy in December 2015. Anecdotal evidence suggests that after the change of family planning policy, many nulliparous women are trying to give vaginal birth in consideration of future pregnancies. However, our study showed that CS on patient request is still very common, accounting for 10.7% of all CS. Although this proportion has declined substantially comparing to previous reports^8^, it is still a major contributor to the high CS rate in Shanghai.

We found that repeat CS has actually become the leading cause for the high CS rate in Shanghai. This is mainly because CS has been popular in China in the past 20 years^9^, resulting in a high proportion of multiparas with a scarred uterus. The recent change in family planning policy to allow two children per family may exacerbate this situation. As more women with previous CS than ever become pregnant and 96.6% of them chose to have repeat CS, the CS in multiparous women may actually increase. Despite that the CS rate in nulliparas could decline, repeat CS may counterbalance. Consequently, the total CS rate may remain unchanged or even increase.

It should be noted that although numerous studies have demonstrated that vaginal birth after previous CS (VBAC) is a safe alternative to repeat CS in carefully selected patients^10^, the urgency of intervention in patients undergoing a trial of labor needs to be in high alert as the avoidance of emergency CS might pose risks for mother and fetus, and increase requirements of general anesthesia and problems in futures pregnancy^3^. Thus, mastering and understanding the indications and contraindications of the trial of labour after caesarean (TOLAC) was the key to success^11^.

Abnormal fetal heart rate was the second leading indication for CS in Shanghai. Sixteen percent of CS deliveries were reported to be due to “fetal heart rate abnormality or fetal distress”. The routine use of continuous fetal heart rate monitoring perhaps permitting longer 2^nd^ stage of labor as long as both progress in descent was being made and fetal safety were assured^12^. However, the routine use of cardiotocography for low-risk women on entrance to the labor ward has been associated with an increase in CS rates and no improvement in perinatal outcomes^13^. The poor sensitivity and specificity of electronic fetal heart rate monitoring often led to false positives when predicting fetal abnormalities. In addition, physician’s judgement on the fetal electrocardiogram is often subjective. Therefore, standardized training for obstetricians and reducing CS based on erroneous judgement play a vital role.

Birth weight is an important determinant of mode of delivery^5^. In our study, the CS rate increased consistently with each 500 g increase in neonatal birth weight above 2500 g in term singleton vertex women in spontaneous labor and pregnant women with a previous CS. For each birth-weight category, the CS rate were higher in nulliparous/multiparous, term singleton vertex women in induced labour (NI/MI group) than in spontaneous labor (NS/MS group). This was consistent to other research results^5^.

In Shanghai, the CS rate was 6.9% in nulliparous, term singleton vertex women in spontaneous labor (NS groups) and the corresponding CS rate was 3.1% in the tertiary maternity hospitals. These seemingly very low rates may be attributable to the very high prelabor CS rate, i.e., only women with good conditions for labor had a trial of labor. These findings are consistent with that in a Brazil study where the corresponding CS rate was 6.3% when neonatal birth weight in 3000 g–3499 g^5^.

Our study has two limitations. First, we have not expert review of each CS record for the information of the underlying circumstances and indications for cesarean section. A more detailed secondary analysis, of the underlying circumstances and indications for cesarean section is needed to operationally identify possible remedial measures in modifiable groups which can reduce the caesarian section rates. Second, we have not investigated the maternal and fetal morbidity and mortality, and their relationship with delivery mode and RTGCS due to lack of detailed clinical information on the causes of CS. Previous studies showed that the rate of CS was positively associated with severe maternal and fetal morbidity and mortality, even after adjustment for risk factors^14,15^. This finding should be confirmed in the future.

In summary, CS rate was still high in Shanghai. Nulliparous women in low risk (with term, term singleton vertex) and having CS before labor, often without any medical indication, was a major contributor to the high CS rate. The tertiary maternity hospital had the lowest CS rate in nulliparous women. Our finding may help us to understand the target for reducing CS rate in Shanghai, China.

## Supplementary information

Indications of caesarean section in different hospital types in Shanghai

## Data Availability

The data are not available freely. However, the datasets from the current study are available from the corresponding author on reasonable request. Clinical trial registration number ChiCTR-IOR-16009041. Date of registration 2016-08-17. Patient consent No informed consent was obtained from individual patients because this study extracted de-identified routine clinical information from medical records.

## References

[1] Ye J (2016). Association between rates of caesarean section and maternal and neonatal mortality in the 21st century: a worldwide population-based ecological study with longitudinal data. BJOG.

[2] Li HT (2017). Geographic Variations and Temporal Trends in Cesarean Delivery Rates in China, 2008-2014. JAMA.

[3] Robson MS (2001). Classification of caesarean sections. Fetal and Maternal Medicine Review.

[4] Torloni MR (2011). Classifications for cesarean section: a systematic review. PLoS One.

[5] Walsh JM, Hehir MP, Robson MS, Mahony RM (2015). Mode of delivery and outcomes by birth weight among spontaneous and induced singleton cephalic nulliparous labors. International Journal of Gynaecology & Obstetrics the Official Organ of the International Federation of Gynaecology & Obstetrics.

[6] W. H. Organization. (Sydney: Health Communications Australia, 2000).

[7] Zhang J (2016). Caesarean section rates in subgroups of women and perinatal outcomes. BJOG.

[8] Zhang J (2008). Cesarean delivery on maternal request in southeast China. Obstet Gynecol.

[9] Hellerstein S, Feldman S, Duan T (2015). China’s 50% caesarean delivery rate: is it too high?. BJOG.

[10] Cunningham FG (2010). NIH consensus development conference draft statement on vaginal birth after cesarean: new insights. NIH Consens State Sci Statements.

[11] He L, Chen M, He GL, Liu XX (2016). Clinical study on vaginal birth after cesarean. Chinese Journal of Obstetrics and Gynecology.

[12] Cohen W (1977). Influence of the duration of second stage labor on perinatal outcome and puerperal morbidity. Obstet Gynecol.

[13] Ayres-de-Campos D, Spong CY, Chandraharan E (2015). FIGO consensus guidelines on intrapartum fetal monitoring: Cardiotocography. Int J Gynaecol Obstet.

[14] Villar J (2006). Caesarean delivery rates and pregnancy outcomes: the 2005 WHO global survey on maternal and perinatal health in Latin America. Lancet.

[15] Lagana, A. S. *et al*. Uterine Scar Healing After Cesarean Section: Managing an Old Surgery in an Evidence-Based Environment. *Journal of investigative surgery: the official journal of the Academy of Surgical Research*, 1–3 (2018). 10.1080/08941939.2018.1465145 29741973

