# Supplementary material for: Dissecting the current caesarean section rate in Shanghai, China
Source: Sci Rep. 2019 Feb 14;9:2080. doi: 10.1038/s41598-019-38606-7 (PMC6376037; doi:10.1038/s41598-019-38606-7)
Supplement: Supplementary file 1 — Indications of caesarean section in different hospital types in Shanghai [file 41598_2019_38606_MOESM1_ESM.pdf]

Dissecting the current caesarean section rate in Shanghai, China

Yanhong Ming[1,2,3#], Meng Li[3,4#], Fei Dai[4], Rong Huang[3], Jinwen Zhang[3,4], Lin Zhang[5], Ming Qin[6], Liping Zhu[6], Hongping Yu[2,7,8], Jun Zhang[2,3*]

[1]Department of Obstetrics and Gynecology, Xiangya Hospital, Central South University, Changsha, Hunan, 410008, China.

[2] School of Public Health, Guilin Medical University, Guilin, Guangxi, 541004, China

[3] Ministry of Education-Shanghai Key Laboratory of Children's Environmental Health, Xinhua Hospital, Shanghai Jiao Tong University School of Medicine, Shanghai, 200092,China

[4] School of Public Health, Shanghai Jiao-Tong University School of Medicine, Shanghai, 200025, China

[5] Xinhua Hospital, Shanghai Jiao Tong University School of Medicine, 1665 Kongjiang Road, Shanghai, 200092,China

[6] Shanghai Maternal and Child Health Center, Shanghai, 200062, China

[7] Affiliate Cancer Hospital of Guangxi Medical University, Nanning, Guangxi, 530021, China

[8]Department of Epidemiology, School of Public Health,Guangxi Medical University,Nanning, Guangxi,530021, China

[#]These authors are considered as co-first authors.

*This author is considered as corresponding authors.

Correspondence

Jun Zhang: Ministry of Education-Shanghai Key Laboratory of Children's Environmental Health, Xinhua Hospital, Shanghai Jiao Tong University School of Medicine, 1665 Kong Jiang Road, 200092, Shanghai, China, Email: zhangjun@xinhuamed.com.cn, Fax: +86-21-25078875,

Table 1 Indications of caesarean section in different hospital types in Shanghai

	Total				Tertiary maternity Hospital			Secondary maternity Hospital			Tertiary general Hospital			Secondary general Hospital		
Indications of CS	N	Absolute CS rate	Proportion of total CS	Order	Absolute CS rate	Proportion of total CS	Order	Absolute CS rate	Proportion of total CS	Order	Absolute CS rate	Proportion of total CS	Order	Absolute CS rate	Proportion of total CS	Order
1.Repeat CS	7764	12.4	29.8	1	8.3	20.4	1	12.9	32.0	1	12.6	26.2	1	15.3	34.9	1
2.Fetal distress	4354	7.0	16.7	2	7.3	18.1	2	4.6	11.5	3	7.8	16.1	2	9.2	21.0	2
3.Patient request	2770	4.4	10.7	3	3.9	9.6	4	5.6	13.9	2	5.2	10.8	3	3.6	8.2	4
4.Non-cephalic fetal presentation	2476	4.0	9.5	4	4.4	11.0	3	4.4	11.0	4	3.8	7.8	4	3.7	8.4	3
5.Suspected macrosomia	1405	2.2	5.4	5	2.4	6.0	5	2.4	5.9	5	2.6	5.3	5	2.1	4.7	6
6.Cephalopelvic disproportion	1008	1.6	3.9	6	0.2	0.5	16	1.8	4.4	6	1.1	2.3	9	2.7	6.1	5

7.Hypertension/ Preeclampsia	760	1.2	2.9	7	2.0	5.0	7	1.0	2.4	8	1.6	3.4	8	0.9	2.0	7
8.Arrested labor/ dystocia	656	1.1	2.5	8	0.7	1.7	10	1.2	3.0	7	2.2	4.6	6	0.6	1.4	9
9.Placenta previa/vasa praevia	599	1.0	2.3	9	1.3	3.1	8	0.7	1.8	10	1.7	3.5	7	0.8	1.7	8
10.Multiple pregnancy	593	1.0	2.3	10	2.1	5.1	6	0.8	2.0	9	0.9	1.8	10	0.6	1.3	10
11. Failed induction of labor	374	0.6	1.4	11	1.0	2.5	9	0.6	1.5	11	0.6	1.2	13	0.4	1.0	11
12. Premature rupture of fetal membranes	264	0.4	1.0	12	0.3	0.6	14	0.3	0.7	14	1.0	2.2	11	0.4	0.9	13
13. Meconium stain	259	0.4	1.0	13	0.5	1.3	11	0.5	1.3	12	0.2	0.5	15	0.4	0.9	12

14. Intrahepatic cholestasis of pregnancy	207	0.3	0.8	14	0.5	1.1	12	0.5	1.2	13	0.4	0.8	14	0.1	0.3	14
15. Diabetes	167	0.3	0.6	15	0.4	1.0	13	0.2	0.6	15	0.6	1.3	12	0.05	0.1	19
16. Heart disease	86	0.1	0.3	16	0.3	0.6	15	0.09	0.2	17	0.2	0.5	17	0.08	0.2	16
17. Placental abruption	69	0.1	0.3	17	0.1	0.3	17	0.09	0.2	16	0.2	0.5	16	0.08	0.2	15
18. Umbilical cord prolapse	34	0.05	0.1	18	0.05	0.1	19	0.03	0.07	20	0.1	0.2	18	0.05	0.1	17
19. Chorioamnionitis	17	0.03	0.07	19	/	/	/	0.03	0.07	18	/	/	/	0.05	0.1	18
20. HIV	11	0.02	0.04	20	0.05	0.1	18	0.03	0.07	19	/	/	/	/	/	/
21. Fetal growth restriction (FGR)	11	0.02	0.04	21	/	/	/	/	/	/	0.1	0.2	19	/	/	/
22. Premature delivery	11	0.02	0.04	22	/	/	/	/	/	/	0.05	0.1	21	0.03	0.07	20

23. Fetal anomaly	5	0.01	0.02	23	/	/	/	/	/	/	0.05	0.1	20	/	/	/
24. Other maternal problems	1503	2.4	5.8	24	3.5	8.6	20	1.8	4.4	21	3.8	7.8	22	1.9	4.4	21
25. Other fetal problems	472	0.8	1.8	25	1.1	2.7	21	0.6	1.5	22	0.9	1.8	23	0.7	1.6	22
26.Other emergency	57	0.09	0.2	26	0.1	0.3	22	0.06	0.2	23	0.2	0.5	24	0.05	0.1	23
27.Unknown	74	0.1	0.3	27	0.2	0.4	23	0.1	0.2	24	0.2	0.4	25	0.1	0.3	24
